# Addressing Social Communication Needs of Dementia Carers: A Feasibility Trial of the CareEngage Program

**DOI:** 10.1093/geroni/igaf122.174

**Published:** 2025-12-31

**Authors:** Raksha Mudar, Lizzy Lydon, Wendy Rogers, Minakshi Raj

**Affiliations:** University of Illinois Urbana-Champaign, University of Illinois Urbana-Champaign, Illinois, United States; Miami University, Oxford, Ohio, United States; University of Illinois Urbana-Champaign, Champaign, Illinois, United States; University of Illinois Urbana-Champaign, Urbana, Illinois, United States

## Abstract

Family carers of persons with dementia (PwD) often experience a decline in meaningful and engaging social communication as caregiving demands increase leading to loneliness, and negative health outcomes. Traditional support groups play an important role in assisting carers with the practical challenges of caregiving. However, they often overlook carers’ broader social communication needs to engage with others in ways that are not centered on caregiving. Speech-language pathologists (SLPs), as healthcare professionals, are uniquely equipped to create person-centered programs that support social communication in carers leveraging their expertise in communication and cognitive rehabilitation, and working in collaboration with interdisciplinary teams. In this study, we developed and optimized a technology-based social engagement program called CareEngage and conducted a feasibility trial using a pragmatic approach with 68 carers of PwD (mean age = 68.8 ± 6.6). Carers joined two weekly, 30-minute Zoom sessions for four weeks, engaging in casual conversations on non-caregiving topics (e.g., Gardening; Healthy Foods). They provided feedback immediately after each session and completed a post-program assessment. The results revealed significantly higher scores on the Friendship Scale (p =.049) and the Social Activity Questionnaire (p =.034) following the program. Session-level and interview data indicated strong satisfaction with social communication with an average enjoyment rating of 4.4 out of 5. These findings suggest that carefully optimized technology-based social engagement programs can support carers’ social communication and health. These results inform healthcare practice and implementation science on developing scalable programs that can be integrated into routine care to improve carer support and outcomes.

